# Job Effort Moderates Associations Between Knowledge Sharing by Chronic Disease Physicians and Patient Health Literacy: Cross-Sectional Study Guided by TPB

**DOI:** 10.1177/00469580261445482

**Published:** 2026-04-25

**Authors:** Renjie Lu, Shaozhuang Ma, Jing Zhou, Jingjie Fan, Qiaoxuan Wu

**Affiliations:** 1 The Affiliated Changzhou Hospital of Xuzhou Medical University, Changzhou Third People’s HospitalChangzhou Third People’s Hospital, Changzhou, Jiangsu, China; 2 ISCTE Business School, BRU-Iscte, University Institute of Lisbon, Lisbon, Portugal; 3 Department of Reproduction, Changzhou Maternal and Child Health Care Hospital, Changzhou Medical Center, Nanjing Medical University, Changzhou, Jiangsu, China; 4 Shenzhen Maternity and Child Healthcare Hospital, Southern Medical University, Shenzhen, China

**Keywords:** Theory of Planned Behavior (TPB), chronic diseases, knowledge sharing, patient health literacy, job effort

## Abstract

**Introduction:**

Effective knowledge sharing by physicians is integral to patient education and health literacy, particularly in the management of chronic diseases. Drawing on the theory of planned behavior (TPB), this study examines how physicians’ attitudes, perceived norms, and perceived behavioral control shape their knowledge-sharing intentions and behaviors, and how these behaviors are associated with patient health literacy within public hospital settings in China.

**Methods:**

A cross-sectional survey of 607 physicians from 39 public hospitals across 6 provinces in China was conducted between March and April 2024. Measures included attitudes, subjective norms, perceived behavioral control, behavioral intention, knowledge-sharing behavior, perceived patient health literacy, and job effort. Structural equation modeling was used to test hypothesized relationships.

**Results:**

Attitudes, subjective norms, and perceived behavioral control were positively associated with physicians’ intentions to share knowledge. Behavioral intention mediated the relationship between perceived behavioral control and actual knowledge-sharing behavior. Actual knowledge-sharing behavior was positively associated with perceived patient health literacy. Importantly, job effort moderated this relationship: high levels of job effort attenuated the positive association between knowledge-sharing behavior and patient health literacy.

**Conclusions:**

Findings underscore the organizational relevance of fostering supportive practice environments that strengthen physicians’ motivation and capacity to share knowledge. Reducing excessive job effort may enhance the impact of knowledge-sharing behaviors on patient health literacy, with implications for hospital management, workforce policy, and chronic disease care delivery.

## Introduction

### Background

Chronic diseases—primarily cardiovascular diseases, cancers, diabetes, and chronic respiratory diseases—are the leading cause of global mortality and disease burden, accounting for 88% of all deaths in China.^1^ According to the World Health Organization, cardiovascular diseases are the leading cause of non-communicable disease (NCD) deaths, responsible for an estimated 17.9 million deaths annually.^2^ Cancer follows with 9 million deaths, chronic respiratory diseases with 3.9 million deaths, and diabetes with 1.6 million deaths.^2^ Collectively, these 4 disease categories account for approximately 80% of all NCD-related deaths worldwide.^2^

China exemplifies this challenge of chronic disease management on a particularly large scale and complexity in the context of rapid demographic and epidemiological transition. According to the National Bureau of Statistics of China (2024), the country has entered a moderately aged society, and population aging is expected to continue.^1^

The burden of chronic disease among older adults is substantial: nationwide evidence indicates that 81% of individuals aged 60 years and above have at least 1 chronic condition.^2^ This burden is further compounded by unhealthy lifestyles, including insufficient physical activity. Given the high treatment costs and disability associated with chronic diseases, these conditions have become a major contributor to escalating healthcare expenditures in China. Therefore, identifying scalable and cost-efficient approaches to chronic disease management has become a pressing public health challenge in China.

Effective management of chronic diseases depends heavily on patient health literacy, yet only about a quarter of the Chinese population possesses basic health knowledge and skills.^3^ Physicians, as authoritative sources of medical information, play a central role in enhancing patients’ health literacy through knowledge sharing.^4^ Research in health communication underscores that effective physician-patient communication is a key pathway for fostering health literacy and improving self-management in chronic illness.^5^

However, several systemic challenges constrain this process. China faces a shortage of physicians and strained physician-patient relationships, resulting in heavy workloads, limited consultation times and mistrust between physicians and patients. In recent years, China’s large population and increasing health awareness have led to an excessive patient load for physicians.^6^ As a result, Chinese physicians’ consultation time is often limited to only 3 to 5 min per patient,^7^ while daily working hours typically exceed 10 hours.^8^ A mixed-methods study comparing physician workloads across countries found that the per capita workload of physicians in China increased drastically by about 42% from 2007 to 2019, far exceeding physician workloads in Europe, Asia, and Australia.^9^ These findings indicate that physicians in China experience substantially higher workloads than their counterparts in many other countries. Physicians in China struggle to fully communicate disease causes, treatment plans, or preventive measures, while patients have little opportunity to express their concerns. Although physicians are driven by professional commitment to patient welfare, time pressure and long workdays—often exceeding 10 h for Chinese physicians may limit their willingness or ability to engage in meaningful knowledge exchange. These conditions raise concerns about how effectively physicians can foster patient health literacy through clinical communication, making China a particularly relevant context for studying factors that influence physicians’ knowledge-sharing behaviors with patients.

To identify targeted opportunities for intervention and ultimately improve both knowledge-sharing behaviors and patient outcomes, we applied the Theory of Planned Behavior (TPB) in this study. TPB posits that behavioral intention, which is the primary predictor of actual behavior, is shaped by attitudes, subjective norms, and perceived behavioral control.^10,11^ Previous studies have applied TPB to examine knowledge-sharing among healthcare professionals. Ryu et al surveyed 286 physicians from 13 tertiary hospitals in South Korea and found that subjective norms had the strongest total effect on physicians’ knowledge-sharing intentions, exerting both a direct effect and an indirect effect through attitude.^12^ Bhatti et al investigated physicians in private and public hospitals in Pakistan and reported that attitude had the largest influence on knowledge-sharing intentions.^13^ These studies demonstrate that TPB provides a valid framework for examining physicians’ knowledge-sharing intentions and behaviors. However, these studies have focused on knowledge sharing among healthcare professionals, with little attention to knowledge sharing from physicians to patients. Examining physician-patient knowledge sharing is particularly important for improving patient health literacy in chronic disease management. Therefore, this study is the first to apply TPB to examine determinants of physicians’ knowledge-sharing intentions and behaviors with chronic disease patients, as well as to assess how such behaviors affect perceived patient health literacy.

In addition, given the demanding work environment, we explore whether job effort moderates these relationships. By linking TPB with health communication scholarship, this study advances understanding of how provider behaviors, contextual pressures, and communication processes jointly shape health literacy.^14^ In doing so, it highlights the importance of creating supportive environments that allow physicians to engage in meaningful knowledge exchange with patients.

### TPB

The TPB proposes that an individual’s attitude, subjective norm, and perceived behavioral control jointly shape behavioral intention, which in turn predicts actual behavior.^10,11^ Attitude refers to one’s positive or negative evaluation of performing a behavior, subjective norm reflects perceived social pressure from others to engage in the behavior, and perceived behavioral control captures the perceived ease or difficulty of performing the behavior, including constraints such as time, resources, or opportunity.^10^ Stronger attitudes, norms, and perceived behavioral control lead to stronger behavioral intentions, which serve as the immediate precursor to actual behavior. Moreover, when individuals perceive sufficient resources and capabilities, perceived behavioral control can also directly influence behavior.^15^

TPB has been widely applied to predict health-related behaviors,^16^ providing a robust framework for understanding the cognitive determinants that shape intentions and actions. However, its application to physician-patient knowledge sharing, particularly from the physicians’ perspective, remains limited. Prior research has largely examined knowledge sharing among healthcare professionals,^17^ often neglecting the critical exchange of knowledge between physicians and patients.

This study extends TPB by examining physicians’ knowledge-sharing intentions and behaviors toward patients and their effects on perceived patient health literacy. By doing so, it provides unique conceptual insights into the determinants of knowledge-sharing behaviors, while offering practical guidance for healthcare organizations seeking to enhance patient outcomes through supportive communication practices and reduced work-related constraints. Understanding these dynamics addresses a notable gap in the literature and highlights the role of physicians in patient education.

### Factors Influencing Knowledge Sharing Behavior

In this study, physicians’ attitudes toward knowledge sharing reflect their positive or negative evaluation of sharing knowledge with patients. Behavioral intention to knowledge sharing refers to the intrinsic motivation of physicians, capturing the effort and time they are willing to invest.^10^ Evidence from healthcare research indicates that attitudes toward knowledge sharing are positively associated with the intention to share knowledge among professionals.^12^ Although TPB has been less applied to physician-patient knowledge sharing, a study on shared decision-making suggests that a positive attitude toward sharing is linked to stronger behavioral intentions.^18^ Accordingly, physicians’ attitudes toward knowledge sharing are expected to influence their intention to share knowledge.

Subjective norm refers to the social pressure physicians perceive regarding knowledge sharing, often arising from patient expectations or professional norms. Subjective norm is a well-established predictor of behavioral intention.^18^ Perceived patient expectations have been identified as barriers to shared decision-making.^18^ These findings suggest that subjective norms can shape physicians’ intention to share knowledge with patients.

Perceived behavioral control captures physicians’ assessment of their ability, resources, and opportunities to successfully share knowledge.^10^ Along with attitude and subjective norm, perceived behavioral control influences behavioral intention and can also directly impact actual behavior.^19^ Among healthcare professionals, perceived behavioral control significantly predicts the intention to share knowledge,^12^ as well as intentions in health education,^20^ and communication.^21^ When physicians perceive sufficient capability and resources, they are more likely to intend to share knowledge and to engage in actual behavior of knowledge sharing. Prior studies suggest that behavioral intention partially mediates the relationship between perceived behavioral control and actual behavior,^22^ although full mediation has also been observed in some contexts.^21^

Grounded in TPB and these empirical findings, we propose the following hypotheses:

***H1a*:** *Attitudes toward knowledge sharing are positively related to behavioral intention to knowledge sharing.*
***H1b*:** *Subjective norms for knowledge sharing are positively related to behavioral intention to knowledge sharing.*
***H1c*:** *Perceived behavioral control for knowledge sharing is positively related to behavioral intention to knowledge sharing.*
***H2a*:** *Perceived behavioral control for knowledge sharing is positively related to actual behavior of knowledge sharing.*
***H2b*:** *Behavioral intention to knowledge sharing is positively related to actual behavior of knowledge sharing.*
***H2c*:** *Behavioral intention to knowledge sharing mediates the relationship between perceived behavioral control for knowledge sharing and actual behavior of knowledge sharing.*

### Outcomes of Knowledge Sharing Behavior: Perceived Patient Health Literacy

In chronic disease management, patient health literacy—the ability to obtain, understand, and use health information—is critical for effective self-management and informed decision-making.^23^ Physicians play a central role in fostering health literacy, not only by providing medical care but also by actively sharing knowledge in ways that patients can comprehend and apply in daily life.^24^

From the perspective of the TPB, physicians’ attitudes, subject norms, and sense of control shape their intentions and actual engagement in knowledge-sharing behaviors.^10,15^ When physicians translate these intentions into actual knowledge-sharing behavior, they directly influence patients’ access to, and understanding of, relevant medical information. Meaningful and accessible communication allows patients to expand their knowledge, better understand treatment options, and take more effective actions to manage their chronic conditions. Based on this reasoning, we hypothesize:

***H3a*:** *Actual behavior of knowledge sharing is positively related to perceived patient health literacy.*

### Sequential Mediation Effects

The preceding hypotheses suggest a process model in which the antecedents of behavior–attitudes toward knowledge sharing, subjective norms for knowledge sharing, and perceived behavioral control for knowledge sharing–shape behavioral intention to knowledge sharing, which in turn influences actual behavior of knowledge sharing. This behavior subsequently impacts perceived patient health literacy, reflecting a sequential mediation structure consistent with recent extensions of the TPB in healthcare contexts.^25^

In this extended TPB framework, perceived patient health literacy is conceptualized as a direct outcome of physicians’ actual behavior of knowledge sharing, a critical factor in chronic disease management where patients’ understanding and engagement are essential for effective long-term care.

Accordingly, perceived patient health literacy functions as a key downstream outcome in a sequential mediation process, whereby behavioral intention to knowledge sharing and actual behavior of knowledge sharing transmit the effects of TPB’s core predictors. Based on this rationale, we propose the following hypotheses:

***H4a*:** *Behavioral intention to knowledge sharing and actual behavior of knowledge sharing sequentially mediate the relationship between attitudes toward knowledge sharing and perceived patient health literacy.*
***H4b*:** *Behavioral intention to share knowledge and actual behavior of knowledge sharing sequentially mediate the relationship between subjective norms for knowledge sharing and perceived patient health literacy.*
***H4c*:** *Behavioral intention to knowledge sharing and actual behavior of knowledge sharing sequentially mediate the relationship between perceived behavioral control for knowledge sharing and perceived patient health literacy.*

### Moderating Role of Job Effort

Job effort refers to the physical, cognitive, and emotional resources that physicians invest in their professional roles, including the time, energy, knowledge, and skills applied during patient interactions. In Chinese public hospitals, physicians often operate under intense time pressure and workload burdens, which can inhibit the translation of intentions into actual behavior of knowledge sharing. Indeed, excessive clinical demands and time constraints have been shown to reduce opportunities for meaningful communication, leading physicians to prioritize efficiency over patient education or to interrupt patients, thereby limiting knowledge exchange and engagement.^26,27^

These conditions suggest that job effort may moderate the effect of actual behavior of knowledge sharing on perceived patient health literacy. Specifically, even when physicians engage in knowledge-sharing, high job effort may reduce the quality or quantity of these interactions, limiting their impact on patients’ health literacy. Based on this reasoning, we hypothesize:

***H5a*:** *Job effort attenuates the positive relationship between physicians’ actual behavior of knowledge sharing and perceived patient health literacy.*

**Figure 1.**
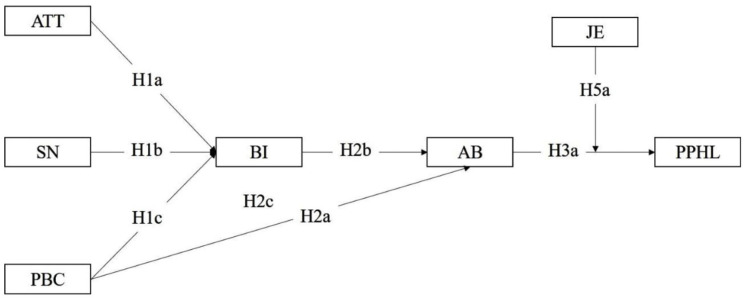
Hypothesized model. *Note.* AB = actual behavior of knowledge sharing; ATT = attitudes toward knowledge sharing; BI = behavioral intention to knowledge sharing; PBC = perceived behavioral control for knowledge sharing; PPHL = perceived patient health literacy; SN = subjective norms for knowledge sharing; H4a = H1a + H2b + H3a; H4b = H1b + H2b + H3a; H4c = H1c + H2b + H3a.

The hypothesized model summarizes the above hypotheses (Figure 1).

## Methods

### Sampling and Procedure

This cross-sectional study surveyed chronic disease physicians across multiple public hospitals in China using a combination of convenience and snowball sampling. In China, chronic disease physicians are primarily employed in public hospitals, work across multiple departments, and typically carry heavy clinical workloads, making them difficult to access for data collection. We employed convenience and snowball sampling to leverage our professional networks to recruit additional physicians, which enabled us to recruit a diverse sample across hospitals and regions.^28,29^ Participation was voluntary, and informed consent was obtained from all respondents prior to data collection.

Hospitals were selected based on their status as public institutions managing chronic disease patients, their willingness to participate, and their capacity to support survey administration. To enhance geographic coverage and include a diverse set of hospitals, initial contacts were made through existing professional and institutional networks, and snowball sampling was subsequently employed to reach additional hospitals. In total, 39 hospitals across 6 provinces (Shanghai, Guangdong, Jiangsu, Zhejiang, Hubei, and Anhui) and 16 cities (eg, Guangzhou, Nanjing, Wuhan, Hangzhou) were included, representing eastern, southern, and central regions of China.

Participants were eligible for inclusion if they (a) were licensed physicians employed in public hospitals in China, (b) were directly involved in the management of patients with chronic diseases, and (c) reported valid data on their average daily outpatient volume and the proportion of patients receiving long-term follow-up care. Physicians were excluded if they (a) practiced in private hospitals, (b) reported a daily outpatient volume of “0” or a “0” proportion of long-term follow-up patients, or (c) were affiliated with departments not related to chronic disease management.

Following standard empirical guidelines, the required sample size was estimated as 10 times the total number of questionnaire items.^30^ Given the likelihood of non-response, several design considerations were implemented to enhance participation. Physicians represent a time-constrained population, and healthcare professionals generally have lower survey response rates than patients.^31^ Moreover, online surveys typically yield lower response rates than in-person administration.^31^ Therefore, questionnaires were administered in person, allowing physicians to complete them at convenient times during their workday and improving response rates and data quality.

Between March 20 and April 30, 2024, a total of 678 paper-based questionnaires were distributed and collected by trained research assistants or designated hospital staff across participating public hospitals in China.

Responses were excluded if any of the following criteria were met: missing hospital name; employment in a private hospital; reported daily outpatient volume or proportion of long-term follow-up patients equal to zero; department unrelated to chronic disease care; incorrect response to an attention-check item (eg, “HIV/AIDS is not an infectious disease”); logical inconsistencies among age, education, and tenure; or uniform responses across all items. After applying these criteria, 607 valid questionnaires (out of 678) remained, yielding an effective response rate of 89.5%.

This study was conducted in accordance with the STROBE (Strengthening the Reporting of Observational Studies in Epidemiology) guidelines for cross-sectional studies,^32^ and the completed checklist is provided as Supplemental File 1.

### Ethical Considerations

The study was approved by the ethics committee of Changzhou Third People’s Hospital (No.02A-A2023032), and all participants’ informed consent was obtained online before the survey. All procedures adhered to the ethical standards outlined in the Declaration of Helsinki.

### Measurement

The full questionnaires, including context-specific modifications, were provided as Supplemental File 2. The demographic and professional information include department, daily outpatient volume, proportion of long-term follow-up patients, among others.

Unless otherwise noted, all items were rated on a 6-point Likert scale ranging from 1 (“strongly disagree”) to 6 (“strongly agree”). Cronbach’s alpha coefficients for all constructs exceeded .70, indicating acceptable internal consistency (Supplemental Table S1).

A previously validated instrument based on the theory of planned behavior was used to assess attitude toward knowledge sharing, subjective norm, perceived behavioral control, and behavioral intention, adapted from previously validated scales.^12,33^ Attitude was measured with 5 bipolar adjective items (eg, 1 = “very harmful,” 6 = “very beneficial”); a sample item is, “*If I share medical knowledge with my patients, I feel it is very harmful*.” Subjective norm was assessed with 5 items capturing perceived social pressure (eg, “*Most physicians who are important to me believe that I should share medical knowledge with my patients*.”). Perceived behavioral control was measured with items evaluating the perceived ease or difficulty of sharing knowledge (eg, “*For me, sharing medical knowledge with my patients is always possible*.”). Behavioral intention was assessed with items reflecting motivation and planning (eg, “*I always plan to share medical knowledge with my patients*.”).

Actual knowledge-sharing behavior was measured using the information-giving dimension of the Medical Communication Competence Scale developed by Cegala et al.^34^ A sample item is, “*I provided clear explanations regarding the causes of my patient’s medical problem*.”

The perceived patient health literacy measurement was informed by previous literature^35^ and adapted to our study setting. The scale aims to provide a comprehensive measure of how physicians perceive their patients’ health literacy levels. A sample item is, “*I believe my patient possesses adequate health knowledge to manage their condition*.”

Job effort was measured with a 3-item scale developed by Siegrist et al^36^ and validated in Chinese samples by Li et al.^37^ A sample item is, “*I experience constant time pressure due to a heavy workload*.”

### Analysis

Descriptive statistics and correlations were conducted using SPSS 26.0. Hypotheses were tested with structural equation modeling (SEM) in Mplus 8.3.^38^ Confirmatory factor analysis (CFA) was performed to assess the measurement model using the maximum likelihood (ML) estimator, which is the default estimation method for continuous indicators. It was conducted to evaluate the construct validity of the measurement model. Convergent and discriminant validity were further assessed based on the average variance extracted (AVE). All constructs demonstrated satisfactory reliability and validity. SEM was then used to examine the relationships among TPB antecedents, actual behavior of knowledge sharing, perceived patient health literacy, and the moderating role of job effort. Model fit was evaluated using standard indices, including χ^2^/df, GFI, AGFI, SRMR, and RMSEA, all indicating acceptable fit.

## Results

### Demographic and Professional Characteristics

As is shown in Table 1, of the 607 participants, 57.8% were female, 83.0% were married, and 75.8% held a master’s degree or higher. Age was evenly distributed across 4 groups: ≤33 (28.0%), 34 to 37 (23.2%), 38 to 42 (24.2%), and ≥43 (24.5%). The tenure of the participants was balanced, with each tenure group representing between 21.7% and 29.5%. In terms of professional titles, attending physicians and associate chief physicians were slightly more prevalent, accounting for 37.2% and 31.3%, respectively. Regarding workload, 40.1% of physicians reported seeing over 50 patients per day, and 44.0% indicated that long-term follow-up patients comprised more than 41.0% of their caseload. Physicians working in hospitals with ≤1000 beds, whether authorized or staffed beds, also made up the largest proportion, representing 33.9% and 33.3%, respectively. Most of the physicians are from the departments of cardiology, respiratory medicine, and endocrinology, with a proportion of 24.2%, 21.4%, and 21.8%, respectively.

**Table 1.** Demographic and Professional Characteristics of Chronic Disease Physicians in the study.

Variables	Options	Frequency	Percentage (%)
Gender	Male	256	42.2
	Female	351	57.8
Age (years)	≤33	170	28.0
	34-37	141	23.2
	38-42	147	24.2
	≥43	149	24.5
Tenure (years)	≤6	154	25.4
	7-12	179	29.5
	13-18	132	21.7
	≥19	142	23.4
Marital status	Married	504	83.0
	Single	102	16.8
	Other (eg, divorced)	1	0.2
Education level	Bachelor’s degree	147	24.2
	Master’s degree	358	59.0
	Doctorate	102	16.8
Professional title	Resident physician	102	16.8
	Attending physician	226	37.2
	Associate chief physician	190	31.3
	Chief physician	89	14.7
Outpatient volume (number of visits)	≤30	212	34.9
	31-50	152	25.0
	51-70	106	17.5
	≥71	137	22.6
Proportion of long-term follow-up patients (%)	≤20	192	31.6
	21-40	146	24.1
	41-60	148	24.4
	≥61	121	19.9
Number of authorized beds	≤1000	206	33.9
	1001-1200	101	16.6
	1201-2050	156	25.7
	≥2051	144	23.7
Number of staffed beds	≤1000	202	33.3
	1001-1500	163	26.9
	1501-2644	114	18.8
	≥2645	128	21.1
Department	Cardiology	147	24.2
	Respiratory medicine	130	21.4
	Endocrinology	132	21.8
	Other chronic diseases	198	32.6

### Correlations

As shown in Table 2, attitudes toward knowledge sharing, subjective norms for knowledge sharing, and perceived behavioral control for knowledge sharing were positively related to behavioral intention to knowledge sharing. Both behavioral intention to knowledge sharing and perceived behavioral control for knowledge sharing were positively associated with actual behavior of knowledge sharing. Actual behavior of knowledge sharing was positively related to perceived patient health literacy. Job effort showed negative associations with attitudes toward knowledge sharing and perceived patient health literacy, suggesting that high job effort may limit knowledge-sharing effectiveness. These findings provide preliminary empirical support for the hypothesized relationships in the TPB-based model.

**Table 2.** Correlations, Means and Standard Deviation.

Variables	Mean (S.D.)	ATT	SN	PBC	BI	AB	PPHL	JE
ATT	5.327 (0.701)	***(.875)***						
SN	5.111 (0.574)	.467^**^	***(.864)***					
PBC	4.864 (0.713)	.433^**^	.429^**^	***(.750)***				
BI	5.003 (0.733)	.542^**^	.577^**^	.454^**^	***(.877)***			
AB	4.638 (0.841)	.356^**^	.475^**^	.305^**^	.524^**^	***(.885)***		
PPHL	3.834 (0.832)	.251^**^	.279^**^	.249^**^	.274^**^	.310^**^	***(.882)***	
JE	4.591 (1.013)	−.130^**^	−.078	−.013	−.040	−.039	−.148^**^	***(.887)***

*Note.* AB = actual behavior of knowledge sharing; ATT = attitudes toward knowledge sharing; BI = behavioral intention to knowledge sharing; PBC = perceived behavioral control for knowledge sharing; PPHL = perceived patient health literacy; SN = subjective norms for knowledge sharing; Cronbach’s alpha α is reported in parenthesis.

** *P* < .01.

### Hypothesized Model Testing

The hypothesized model was tested using Mplus 8.3. The structural equation model showed an acceptable fit (χ^2^ = 1272, df = 315, χ^2^/df = 4.04, CFI = 0.905, TLI = 0.894, RMSEA = 0.071, SRMR = 0.073), supporting the adequacy of the proposed model.^39,40^ To ensure robustness, we conducted 5000 bootstrap resamples to estimate confidence intervals for key paths.

Based on the hypothesized model, we tested the proposed relationships (Table 3). Results indicated that attitudes toward knowledge sharing were positively associated with behavioral intention to knowledge sharing (β = .254, *P* < .001), supporting H1a. Similarly, subjective norms for knowledge sharing positively predicted behavioral intention to knowledge sharing (β = .303, *P* < .001), supporting H1b, and perceived behavioral control for knowledge sharing was also positively related to behavioral intention to knowledge sharing (β = .322, *P* < .001), supporting H1c.

**Table 3.** Estimated Path Coefficients for Structural Equation Model Examining Knowledge Sharing and Patient Health Literacy.

Outcome	Predictor	B	SE	B/SE	*P*	95% CI	
						Lower	Upper
BI	ATT	0.254	0.052	4.843	<.001	0.151	0.357
BI	SN	0.303	0.058	5.234	<.001	0.190	0.416
BI	PBC	0.322	0.062	5.216	<.001	0.201	0.443
AB	BI	0.483	0.058	8.277	<.001	0.369	0.597
AB	PBC	0.144	0.063	2.295	.022	0.021	0.267
PPHL	AB	0.303	0.043	7.064	<.001	0.219	0.388

*Note.* AB = actual behavior of knowledge sharing; ATT = attitudes toward knowledge sharing; BI = behavioral intention to knowledge sharing; PBC = perceived behavioral control for knowledge sharing; PPHL = perceived patient health literacy; SN = subjective norms for knowledge sharing.

Both perceived behavioral control for knowledge sharing and behavioral intention to knowledge sharing were positively associated with actual behavior of knowledge sharing (β = .144, *P* < .05; β = .483, *P* < .001), supporting H2a and H2b. Furthermore, actual behavior of knowledge sharing significantly predicted perceived patient health literacy (β = .303, *P* < .001), providing support for H3a.

### Mediation and Sequential Mediation Effects

As shown in Table 4, behavioral intention to knowledge sharing mediated the relationship between perceived behavioral control for knowledge sharing and actual behavior of knowledge sharing. The indirect effect of perceived behavioral control for knowledge sharing → behavioral intention to knowledge sharing → actual behavior of knowledge sharing was significant (β = .155, *P* < .001), while the direct effect of perceived behavioral control for knowledge sharing → actual behavior of knowledge sharing remained significant (β = .144, *P* = .022), indicating partial mediation and supporting H2c.

**Table 4.** Direct, Indirect, and Total Effects of Behavioral Intention as a Mediator Between Perceived Behavioral Control and Actual Knowledge Sharing Behavior.

Effect	Path	β	SE	*P*	95% CI	
					Lower	Upper
Indirect	PBC->BI->AB	.155	0.034	<.001	0.089	0.222
Direct	PBC->AB	.144	0.063	.022	0.021	0.267
Total	PBC->AB	.300	0.056	<.001	0.189	0.410

*Note.* AB = actual behavior of knowledge sharing; ATT = attitudes toward knowledge sharing; BI = behavioral intention to knowledge sharing; PBC = perceived behavioral control for knowledge sharing; PPHL = perceived patient health literacy; SN = subjective norms for knowledge sharing.

Sequential mediation effects were further examined to evaluate the mechanisms linking TPB antecedents to perceived patient health literacy through behavioral intention to knowledge sharing and actual behavior of knowledge sharing (Table 5).

**Table 5.** Mediation Effects of the Baseline Structural Equation Model Examining Knowledge Sharing and Patient Health Literacy.

Predictor	Mediator	Outcome	B	SE	β/SE	*P*	95% CI	
							Lower	Upper
ATT	BI	AB	0.123	0.030	4.090	<.001	0.064	0.181
SN	BI	AB	0.146	0.036	4.048	<.001	0.075	0.217
PBC	BI	AB	0.155	0.034	4.585	<.001	0.089	0.222
ATT	AB, BI	PPHL	0.037	0.010	3.679	<.001	0.017	0.057
SN	AB, BI	PPHL	0.044	0.013	3.415	.001	0.019	0.070
PBC	AB	PPHL	0.044	0.021	2.102	.036	0.003	0.085
PBC	AB, BI	PPHL	0.047	0.013	3.761	<.001	0.023	0.072

*Note.* AB = actual behavior of knowledge sharing; ATT = attitudes toward knowledge sharing; BI = behavioral intention to knowledge sharing; PBC = perceived behavioral control for knowledge sharing; PPHL = perceived patient health literacy; SN = subjective norms for knowledge sharing.

- Attitudes toward knowledge sharing: Attitudes toward knowledge sharing indirectly influenced actual behavior via behavioral intention to knowledge sharing (β = .123, *P* < .001) and indirectly affected perceived patient health literacy through behavioral intention to knowledge sharing and actual behavior of knowledge sharing (β = .037, *P* < .001), supporting H4a.
- Subjective norms for knowledge sharing: Subjective norms for knowledge sharing indirectly affected actual behavior of knowledge sharing via behavioral intention to knowledge sharing (β = .146, *P* < .001) and perceived patient health literacy via behavioral intention to knowledge sharing and actual behavior of knowledge sharing (β = .044, *P* = .001), supporting H4b.
- Perceived behavioral control for knowledge sharing: Perceived behavioral control for knowledge sharing indirectly influenced actual behavior of knowledge sharing through behavioral intention to knowledge sharing (β = .155, *P* < .001), and perceived patient health literacy through behavioral intention to knowledge sharing and actual behavior of knowledge sharing (β = .047, *P* < .001). Perceived behavioral control for knowledge sharing also had a smaller indirect effect on perceived patient health literacy via actual behavior of knowledge sharing alone (β = .044, *P* = .036), supporting H4c.

These results demonstrated that behavioral intention to knowledge sharing and actual behavior of knowledge sharing jointly transmit the effects of TPB antecedents on both actual behavior of knowledge sharing and perceived patient health literacy.

### Moderation Effect of Job Effort

**Figure 2.**
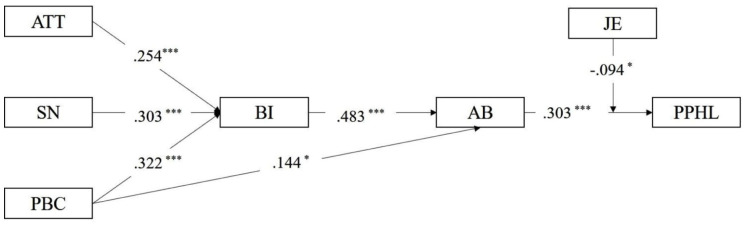
Path diagram of hypothesis model. *Note.* AB = actual behavior of knowledge sharing; ATT = attitudes toward knowledge sharing; BI = behavioral intention to knowledge sharing; JE = job effort; PBC = perceived behavioral control for knowledge sharing; PPHL = perceived patient health literacy; SN = subjective norms for knowledge sharing. *P < .05. **P < .01. ***P < .001.

The interaction between job effort and actual behavior of knowledge sharing was significant (β = −.094, *P* = .017), indicating that job effort attenuated the positive relationship between actual behavior of knowledge sharing and perceived patient health literacy (Figure 2).

**Figure 3.**
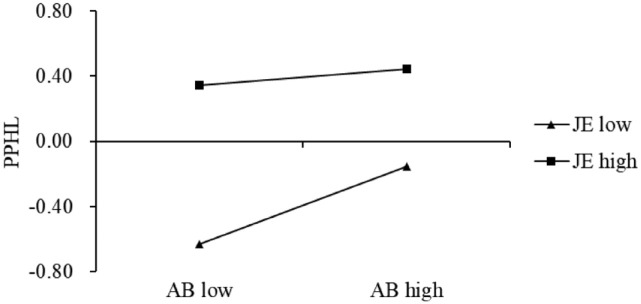
Interaction effect slope graph. *Note.* AB = actual behavior of knowledge sharing; JE = job effort; PPHL = perceived patient health literacy.

A slope analysis was conducted to illustrate the moderating effect of job effort on the relationship between actual behavior of knowledge sharing and perceived patient health literacy (Figure 3). When job effort was low, actual behavior of knowledge sharing was strongly and positively associated with perceived patient health literacy. Under high job effort, the slope decreased, indicating a weaker positive relationship. These results supported H5a.

## Discussion

This study applied the TPB to examine knowledge-sharing among physicians treating chronic diseases and its effects on perceived patient health literacy, with job effort as a moderating factor. The findings generally support TPB’s central propositions: Attitudes toward knowledge sharing and subjective norms for knowledge sharing strengthened physicians’ behavioral intention to knowledge sharing, consistent with prior work highlighting the reinforcing role of peer recognition and organizational expectations.^41^ Perceived behavioral control for knowledge sharing was positively associated with both behavioral intention to knowledge sharing and actual behavior of knowledge sharing, suggesting that competence, communication skills, and time management facilitate the translation of willingness into action.^42^ As TPB posits, behavioral intention to knowledge sharing predicted actual behavior of knowledge sharing.^33^ These findings demonstrate that the TPB’s central mechanisms operate in the physician-patient knowledge-sharing context.

Actual behavior of knowledge sharing was positively associated with perceived patient health literacy, supporting evidence that patient education improves understanding and self-management in chronic care.^43^ Therefore, in the context of chronic disease management, physicians’ knowledge-sharing behavior plays a beneficial role: it positively predicts patient health literacy.

The results also showed that behavioral intention to knowledge sharing only partially mediated the relationship between perceived behavioral control for knowledge sharing and actual behavior of knowledge sharing, which aligns with TPB’s distinction between perceived and actual behavioral control, as intentions can only translate into behavior when sufficient actual control exists.^11^ This indicates that even when physicians intend to share knowledge, external constraints such as limited time or resources may prevent intentions from fully translating into action. As Bosnjak, Ajzen and Schmidt note, intention predicts behavior most reliably when actual control is sufficient.^44^ This points to the importance of supportive organizational conditions that allow physicians to act on their intentions.

A sequential pathway emerged from attitudes toward knowledge sharing, subjective norms for knowledge sharing, and perceived behavioral control for knowledge sharing through behavioral intention to knowledge sharing and actual behavior of knowledge sharing to perceived patient health literacy. Specifically, physicians’ positive attitudes toward knowledge sharing and perceived social expectations increased their intention to share knowledge, and higher perceived control facilitated the translation of intention into actual behavior of knowledge sharing, which in turn enhanced patients’ perceived health literacy, directly reflecting the mechanisms proposed by TPB.^44^ While this aligns with TPB, past studies have reported variation in the strength of these links. In some contexts, subjective norms exert less influence when professional autonomy dominates decision-making,^45^ whereas in more collaborative environments, norms can play a stronger role.^18^ Similarly, intention does not always lead to behavior in health communication, where competing demands and systemic pressures often intervene.^46^ The present findings thus fit within the broader literature while also highlighting the conditions under which TPB predictions hold.

Notably, job effort weakened the relationship between actual behavior of knowledge sharing and perceived patient health literacy. Heavy workloads, time pressure, and frequent interruptions limit physicians’ ability to communicate effectively, diminishing the benefits for patients.^24^ Theoretically, this finding highlights a limitation of TPB: while it captures motivational and normative drivers of behavior, it pays less attention to contextual constraints. In practice, this means that fostering knowledge-sharing intentions and behavior is necessary but not sufficient-reducing workload and creating supportive structures are also critical to ensure that knowledge-sharing translates into patient benefits.

## Implications

### Theoretical Contributions

This study applies the Theory of Planned Behavior (TPB) to elucidate the mechanisms underlying physicians’ knowledge-sharing and advances the literature in 3 interrelated ways.

First, it extends TPB-based research by integrating intention, behavior, contextual constraints, and patient-level outcomes within a single analytical framework. Specifically, we examine both knowledge-sharing intention and actual behavior, test job effort as a boundary condition on the intention–behavior relationship, and link physicians’ knowledge-sharing to a patient-relevant outcome—perceived patient health literacy. By connecting cognitive antecedents, organizational context, and patient impact, the study moves beyond intention-focused models and provides a more comprehensive account of how knowledge-sharing translates into meaningful healthcare outcomes.

Second, the study introduces job effort as a theoretically relevant moderator, thereby contextualizing TPB within high-demand healthcare environments. By modeling job effort as a constraint that may attenuate the intention–behavior link, we add an organizational dimension to TPB and demonstrate how workload conditions shape the enactment of prosocial professional behaviors. This contributes to the refinement of TPB by identifying boundary conditions under which intentions are less likely to translate into action, highlighting the complexity of physicians’ behavior in resource-constrained settings.

Third, by focusing on physicians in China’s public hospitals—characterized by heavy workloads and escalating chronic disease demands—we provide evidence from a high-pressure healthcare context that remains underrepresented in the knowledge-sharing literature. This empirical setting enables a robust test of TPB in a non-Western, systemically strained environment and offers comparative insight for international research. By incorporating perceived patient health literacy as an outcome, the study further positions knowledge-sharing as a mechanism linking physician behavior to patient empowerment, reinforcing the theoretical and practical relevance of TPB for health communication and chronic disease management research.

### Managerial Implications

From a practical and policy standpoint, our findings provide implications to address the challenges posed by population aging and the rapid rise of chronic diseases in China. Our results identify modifiable determinants of physicians’ knowledge-sharing behavior and highlight structural barriers (eg, high job effort) that may impede effective physician’s knowledge-sharing. This provides actionable guidance for hospital administrators and policymakers seeking to strengthen chronic care models, improve physician–patient communication, and enhance patient health literacy—an essential component of long-term disease control.

Specifically, healthcare organizations can foster a culture that values knowledge sharing by shaping positive attitudes and reinforcing supportive norms. In contexts such as China, where professional traditions discourage open exchange, training and educational initiatives can emphasize the benefits of sharing for both patient outcomes and professional growth. Encouraging open exchange, framing information in patient-centered ways, and modeling effective communication can gradually shift attitudes and norms in clinical practice. Senior physicians play a critical role in modeling these behaviors, and recognition systems-such as incorporating knowledge-sharing into promotion criteria or performance evaluations-can motivate others to follow suit.

Organizational conditions are equally important for enabling physicians to facilitate the process from knowledge sharing to patient benefits. Heavy workloads, time pressure, and limited consultation times often restrict opportunities for meaningful communication and knowledge sharing. Hospital administrators and health authorities should prioritize reducing these barriers and providing adequate resources for patient education. Leveraging health information technologies, such as electronic health records, can also support efficient and streamlined communication between physicians and patients.

Finally, strengthening physicians’ sense of control over the communication process is crucial. Training that emphasizes patient-centered communication and knowledge-delivery strategies can enhance this perceived control. Digital tools-including mobile health applications, hospital platforms, or AI-assisted education modules-can also support physicians by providing patients with foundational information before or after consultations. When patients arrive better informed, physicians can devote consultation time to more personalized, targeted exchanges that improve both efficiency and patient health literacy.

## Limitations and Future Research

At the same time, these interpretations should be viewed within the methodological boundaries of the present study. First, the cross-sectional design allows identification of robust associations but does not permit causal inference regarding the directionality of knowledge sharing, job effort, and patient health literacy. Second, the reliance on physicians’ self-reported assessments may have inflated observed relationships due to common method variance and social desirability bias. Third, the absence of patient-reported or behavioral outcome measures constrains conclusions about whether knowledge-sharing behaviors result in observable improvements in patient literacy or self-management. Furthermore, although this study recruited physicians from 39 public hospitals across 16 cities in 6 provinces, the use of convenience and snowball sampling may have introduced selection bias and limited representation of physicians outside the reachable networks. The study did not encompass all regions or international healthcare settings, which may limit the generalizability of the findings across different health systems and cultural contexts.

Given these limitations, the generalizability of the findings to other medical specialties or healthcare systems warrants further investigation. In addition, as the study relied exclusively on quantitative methods, it may not fully capture the nuanced and dynamic processes underlying physician-patient knowledge sharing. Future research could incorporate unmeasured contextual and organizational factors, such as departmental culture, institutional policies, patient engagement, and social support, and employ qualitative or mixed-methods approaches to provide deeper insight into these mechanisms. Such studies would also help further validate the applicability of TPB in diverse clinical and cultural contexts.

## Conclusions

This study confirms the explanatory utility of the TPB in understanding physicians’ knowledge-sharing behaviors while also delineating its practical boundaries. It is the first to apply TPB to physician–patient knowledge sharing in chronic disease management in China, extending prior research that has largely focused on knowledge exchange among healthcare professionals. The findings demonstrate that attitudes, subjective norms, perceived behavioral control, and job effort jointly shape physicians’ knowledge-sharing intentions and their actual communication behaviors with patients.

Practically, the results underscore that both individual-level factors (eg, attitudes, perceived control, skills, effort) and organizational conditions influence effective knowledge sharing and, by extension, patient health literacy. Designing knowledge-sharing interventions for chronic disease management therefore requires attention to physicians’ motivational and capability-related determinants, as well as structural constraints that may impede meaningful interaction.

In the context of rapid population aging and rising chronic disease prevalence in China, these findings provide evidence-based guidance for developing targeted, scalable strategies that enhance patient-centered education, and ultimately improve chronic disease self-management and health outcomes among older adults.

## Supplemental Material

sj-docx-1-inq-10.1177_00469580261445482 – Supplemental material for Job Effort Moderates Associations Between Knowledge Sharing by Chronic Disease Physicians and Patient Health Literacy: Cross-Sectional Study Guided by TPB Supplemental material, sj-docx-1-inq-10.1177_00469580261445482 for Job Effort Moderates Associations Between Knowledge Sharing by Chronic Disease Physicians and Patient Health Literacy: Cross-Sectional Study Guided by TPB by Renjie Lu, Shaozhuang Ma, Jing Zhou, Jingjie Fan and Qiaoxuan Wu in INQUIRY: The Journal of Health Care Organization, Provision, and Financing

sj-docx-2-inq-10.1177_00469580261445482 – Supplemental material for Job Effort Moderates Associations Between Knowledge Sharing by Chronic Disease Physicians and Patient Health Literacy: Cross-Sectional Study Guided by TPB Supplemental material, sj-docx-2-inq-10.1177_00469580261445482 for Job Effort Moderates Associations Between Knowledge Sharing by Chronic Disease Physicians and Patient Health Literacy: Cross-Sectional Study Guided by TPB by Renjie Lu, Shaozhuang Ma, Jing Zhou, Jingjie Fan and Qiaoxuan Wu in INQUIRY: The Journal of Health Care Organization, Provision, and Financing
