# Supplementary material for: Job Effort Moderates Associations Between Knowledge Sharing by Chronic Disease Physicians and Patient Health Literacy: Cross-Sectional Study Guided by TPB
Source: Inquiry. 2026 Apr 25;63:00469580261445482. doi: 10.1177/00469580261445482 (PMC13129283; doi:10.1177/00469580261445482)
Supplement: sj-docx-2-inq-10.1177_00469580261445482 – Supplemental material for Job Effort Moderates Associations Between Knowledge Sharing by Chronic Disease Physicians and Patient Health Literacy: Cross-Sectional Study Guided by TPB [file sj-docx-2-inq-10.1177_00469580261445482.docx]

**Appendix Questionnaire**

No：

Instructions for Participants

You are invited to participate in this questionnaire. All responses will be treated with strict confidentiality and used solely for academic research purposes. There are no right or wrong answers; please respond based on your own experiences.

As a **specialist in chronic disease**, please indicate with a “√” the option that best reflects your typical situation. In this questionnaire, the term “**patients**” refers specifically to **long-term follow-up chronic disease patients**. We appreciate your participation and support.

1. Attitude (AT) Toward Knowledge Sharing

Please mark “√” on the option that best reflects your actual experience or perception.

| … share medical knowledge…, I feel it is…… | | | | | | | |
| --- | --- | --- | --- | --- | --- | --- | --- |
| AT1 | 1=Very harmful ~ 6=Very beneficial | 1 | 2 | 3 | 4 | 5 | 6 |
| AT2 | 1=Very unpleasant ~ 6=Very pleasant | 1 | 2 | 3 | 4 | 5 | 6 |
| AT3 | 1=Very bad ~ 6=Very good | 1 | 2 | 3 | 4 | 5 | 6 |
| AT4 | 1=Very worthless ~ 6=Very valuable | 1 | 2 | 3 | 4 | 5 | 6 |
| AT5 | 1=Very unenjoyable ~ 6=Very enjoyable | 1 | 2 | 3 | 4 | 5 | 6 |

2. Subjective Norm (SN) for Knowledge Sharing

Please mark “√” on the option that best reflects your actual experience or perception.

| No | Items | Strongly Disagree | Disagree | Slightly Disagree | Slightly Agree | Agree | Strongly Agree |
| --- | --- | --- | --- | --- | --- | --- | --- |
| SN1 | … expected of me that I … . | 1 | 2 | 3 | 4 | 5 | 6 |
| … who are important to me | | | | | | | |
| SN2 | … think that I should share medical knowledge … . | 1 | 2 | 3 | 4 | 5 | 6 |
| SN3 | … share their medical knowledge … . | 1 | 2 | 3 | 4 | 5 | 6 |
| … whose opinions I value | | | | | | | |
| SN4 | … would approve of my behavior to share medical knowledge … . | 1 | 2 | 3 | 4 | 5 | 6 |
| SN5 | … share their medical knowledge … . | 1 | 2 | 3 | 4 | 5 | 6 |

|  | Strongly Disagree | Disagree | Slightly Disagree | Slightly Agree | Agree | Strongly Agree |
| --- | --- | --- | --- | --- | --- | --- |
| Hypertension is a contagious disease. | 1 | 2 | 3 | 4 | 5 | 6 |

3. Perceived Behavioral Control (PBC) for Knowledge Sharing

Please mark “√” on the option that best reflects your actual experience or perception.

| No | Items | Strongly Disagree | Disagree | Slightly Disagree | Slightly Agree | Agree | Strongly Agree |
| --- | --- | --- | --- | --- | --- | --- | --- |
| PBC1 | … to share my medical knowledge … . | 1 | 2 | 3 | 4 | 5 | 6 |
| PBC2 | … I want, … always could share … . | 1 | 2 | 3 | 4 | 5 | 6 |
| PBC3 | … mostly up to me …. | 1 | 2 | 3 | 4 | 5 | 6 |
| PBC4 | … believe that there are much control I have to ... . | 1 | 2 | 3 | 4 | 5 | 6 |

|  | Strongly Disagree | Disagree | Slightly Disagree | Slightly Agree | Agree | Strongly Agree |
| --- | --- | --- | --- | --- | --- | --- |
| The emergency medical service number in China is 119. | 1 | 2 | 3 | 4 | 5 | 6 |

4. Behavioral Intention (BI) to Share Knowledge

Please mark “√” on the option that best reflects your actual experience or perception.

| No | Items | Strongly Disagree | Disagree | Slightly Disagree | Slightly Agree | Agree | Strongly Agree |
| --- | --- | --- | --- | --- | --- | --- | --- |
| … always will | | | | | | | |
| BI1 | … plan to share … . | 1 | 2 | 3 | 4 | 5 | 6 |
| BI2 | … try to share … . | 1 | 2 | 3 | 4 | 5 | 6 |
| BI3 | … make an effort to share … . | 1 | 2 | 3 | 4 | 5 | 6 |
| BI4 | … intend to share … . | 1 | 2 | 3 | 4 | 5 | 6 |

5. Actual Behavior (AB) of Knowledge Sharing

Please mark “√” on the option that best reflects your actual experience or perception.

| No | Items | Never | Rarely | Sometimes | Often | Frequently | Always |
| --- | --- | --- | --- | --- | --- | --- | --- |
| … explanations regarding | | | | | | | |
| AB1 | … the causes of … . | 1 | 2 | 3 | 4 | 5 | 6 |
| AB2 | … the treatment for my patient’s … . | 1 | 2 | 3 | 4 | 5 | 6 |
| AB3 | … the purpose of any tests that … . | 1 | 2 | 3 | 4 | 5 | 6 |
| AB4 | … how to take … . | 1 | 2 | 3 | 4 | 5 | 6 |
| AB5 | …the long-term consequences of … . | 1 | 2 | 3 | 4 | 5 | 6 |

|  | Strongly Disagree | Disagree | Slightly Disagree | Slightly Agree | Agree | Strongly Agree |
| --- | --- | --- | --- | --- | --- | --- |
| AIDS is not a contagious disease. | 1 | 2 | 3 | 4 | 5 | 6 |

6. Perceived Patient Health Literacy

Please mark “√” on the option that best reflects your actual experience or perception.

| No | Items | Strongly Disagree | Disagree | Slightly Disagree | Slightly Agree | Agree | Strongly Agree |
| --- | --- | --- | --- | --- | --- | --- | --- |
| PPHL1 | … my patients have good knowledge about …. | 1 | 2 | 3 | 4 | 5 | 6 |
| PPHL2 | … have enough knowledge …. | 1 | 2 | 3 | 4 | 5 | 6 |
| PPHL3 | … my patients have all the knowledge …. | 1 | 2 | 3 | 4 | 5 | 6 |
| PPHL4 | … have all the knowledge they need to …. | 1 | 2 | 3 | 4 | 5 | 6 |

7. Job Effort

Please mark “√” on the option that best reflects your actual experience or perception.

| No | Items | Strongly Disagree | Disagree | Slightly Disagree | Slightly Agree | Agree | Strongly Agree |
| --- | --- | --- | --- | --- | --- | --- | --- |
| JE1 | … have constant time pressure due to … . | 1 | 2 | 3 | 4 | 5 | 6 |
| JE2 | … have many interruptions and disturbances … . | 1 | 2 | 3 | 4 | 5 | 6 |
| JE3 | Over the past few years, … has become … . | 1 | 2 | 3 | 4 | 5 | 6 |

8. Demographic and professional characteristics

1) Department: □Cardiology □Respiratory Medicine □Endocrinology □Other:

2) Approximate number of daily outpatient volume: (visits)

3) Approximate proportion of long-term follow-up patients in daily outpatient visits: (%)

4) Gender: □Male □Female

5) Age: (years)

6) Tenure (years): (years)

7) Marital status: □Married □Single □Other (e.g., divorced)

8) Highest education level:

□Bachelor’s degree □Master’s degree □Doctoral degree

9) Professional title:

□Resident physician □Attending physician □Associate chief physician □Chief physician

10) Number of authorized beds: (beds)

11) Number of staffed beds: (beds)

12) Name of hospital:
